# Structural variation analysis suggests strain-level maternal-infant microbial transmission in early life

**DOI:** 10.3389/fcimb.2026.1765801

**Published:** 2026-05-19

**Authors:** Xin Jiang, Bin Chen, Qinghua Wang, Yanan Liu, Ning Li, Lei Zhang

**Affiliations:** 1 Microbiome-X, School of Public Health, Cheeloo College of Medicine, Shandong University, Jinan, China; 2 School of Biological Science and Technology, University of Jinan, Jinan, China; 3 Jinan Institute of Child Health Care, Children's Hospital Affiliated to Shandong University (Jinan Children's Hospital), Jinan, China; 4 School of Pharmaceutical Engineering, Jining Medical University, Jining, China

**Keywords:** early-life development, infant colonization, maternal microbiota, metagenomics, structural variations, vertical transmission

## Abstract

**Introduction:**

Structural variations (SVs)—large, functionally consequential genomic alterations—serve as high-resolution markers for strain-level differentiation in the human microbiome, yet their relevance to vertical transmission of the maternal microbiota and early-life colonization remains unclear.

**Methods:**

Using metagenomic data from a 98-pair longitudinal mother-infant cohort and a 25-pair multi-niche cohort, we profiled microbial taxa, functions, and SVs, characterized variable SVs (vSVs), deletion SVs (dSVs), and transmitted SVs (tSVs), and evaluated the potential influence of delivery mode, feeding regimen, and maternal ecological niches.

**Results:**

We identified 5,578 SVs across 51 reference strains, with infants showing increasing SV diversity during the first year of life, and observed significantly greater SV similarity within mother-infant pairs than unrelated pairs. Abundance-based analysis identified 90 microbial species shared between mothers and infants. However, when incorporating SV-based tracking, only 14 strains showed patterns consistent with sustained maternal contribution across time points. Furthermore, exploratory subgroup analyses suggested that both delivery mode and feeding regimen may influence the vertical transmission patterns of maternal microbial strains and transmitted SVs. Functionally, tSVs were enriched in pathways linked to carbohydrate, amino acid, and lipid metabolism, as well as transport and environmental adaptation modules such as T4SS. Multi-niche analysis further suggested that the maternal gut showed the strongest inferred signal of SV-supported strain sharing with both the infant gut and oral microbiota.

**Discussion:**

Together, these findings suggest that microbial SVs can serve as complementary markers for investigating maternal contribution and vertical transmission-related strain-level patterns in early-life microbiome development, providing new insights into microbial inheritance and early-life health trajectories.

## 1 Introduction

The establishment of the complex microbiota that colonizes the human body and the development of its symbiotic relationship with the host both begin in the perinatal period and are shaped by diverse intrinsic and extrinsic factors (Wang et al., 2021b). Vertical transmission of microorganisms from mother to infant is considered an important process in the establishment of the infant gut microbiota (Milani et al., 2015; Maher et al., 2020). Early maternal microbial colonizers may also influence subsequent microbiota assembly, as well as the dynamic changes and maturation of the microbiota during infant growth (Feehily et al., 2023). Furthermore, the maternal microbiota is crucial for infant development and health, including immune regulation and nervous system development (Ferretti et al., 2018). It has also been associated with the risk of certain diseases, such as diabetes, inflammatory bowel disease, obesity, and asthma (Ladomenou et al., 2010; Klopp et al., 2017). However, whether the genetic makeup of vertically transmitted microbiota is associated with infant development and health remains largely unclear. Characterizing structural variations (SVs) in the context of maternal microbiota vertical transmission may improve our understanding of mother-infant microbial transfer and provide clues for exploring microbiota-related pathways potentially relevant to infant development and disease susceptibility.

Structural variations are large genomic alterations, such as deletions and insertions, that can alter microbial function, potentially leading to strains of the same species with different functional capacities (Wang et al., 2021a). Differences in the genetic makeup of individual strains underlie a spectrum of phenotypic functions, such as nutrient utilization efficiency and pathogenic potential, thereby influencing their effect on the host (Shen et al., 2024). Accordingly, SVs can provide subgenomic resolution information on bacterial functionality (Wang et al., 2021a). Microbial genomic structural variants are prevalent in the human intestinal microbiome (Zeevi et al., 2019). Previous studies have shown that microbial SVs may affect microbial functions (Zhernakova et al., 2024) and are subsequently associated with the occurrence of certain diseases (Zeevi et al., 2019; Wang et al., 2021a; Huang et al., 2022; Liu et al., 2023). Additionally, SVs in some species can persist stably within an individual (Chen et al., 2021).

Therefore, we hypothesized that microbial SVs may provide complementary, strain-level information relevant to mother-infant vertical transmission. We further hypothesized that delivery mode, feeding regimen, and maternal ecological niches may be associated with SV-based strain-level patterns and their persistence across infancy. To explore these questions, we analyzed SVs in two mother-infant cohorts to identify strain-level patterns supporting mother-to-infant vertical transmission, describe the annotated features of SV-associated regions, and evaluate the associations of delivery mode, feeding regimen, and maternal ecological niches with these patterns during early life.

## 2 Materials and methods

### 2.1 Study cohorts

This study was based on a secondary analysis of metagenomic data from two previously established mother-infant cohorts. Participant recruitment and original inclusion/exclusion criteria were defined in the corresponding source studies. For the present analysis, we included datasets that contained mother-infant metagenomic samples relevant to our objectives. The first dataset was a gut metagenomic dataset comprising 98 mother-infant pairs (Gerd et al., 2012; Bäckhed et al., 2015). The infant cohort comprised 44 males and 54 females, all born at full term (37-42 weeks of gestation). Of these, 83 infants were delivered vaginally and 15 by cesarean section. Stool samples were collected from mothers at birth and from infants at birth, 4 months, and 12 months. Infant feeding practices at the corresponding time points were recorded. All samples were stored at −80 °C until sequencing.

The second dataset comprised longitudinal metagenomic data from 25 mother-infant pairs across multiple maternal and infant ecological niches, including maternal fecal, skin, vaginal, and oral samples, as well as infant fecal and oral samples (Ferretti et al., 2018). The study recruited 25 pregnant women aged 26-43 years at a hospital in Italy between 2015 and 2016. The original study was approved by the ethical review committee. Oral samples were collected from infants on the first and third days after birth. Fecal samples were collected from infants on day 1, day 3, day 7, month 1, and month 4 after birth.

### 2.2 Metagenomic data preprocessing

FastQC (v0.11.9) and MultiQC (v1.12) were used to assess the quality of the downloaded raw metagenomic data and summarize the quality-control results (Ewels et al., 2016). Raw paired-end reads were quality-filtered using Trimmomatic (version 0.39) with a sliding-window approach, and reads shorter than 50 bp after trimming were discarded (Bolger et al., 2014). Host-derived reads were removed by aligning filtered reads to the human reference genome hg19 using Bowtie2 (v2.4.1), and only non-host paired reads were retained for downstream analyses (Langmead and Salzberg, 2012).

### 2.3 Taxonomic composition and function

Taxonomic profiles were generated from the quality-controlled metagenomic data of both cohorts using MetaPhlAn3 (v3.0.14), and species-level relative abundance profiles were extracted for downstream analyses (Segata et al., 2012). Functional profiles were generated from the quality-controlled metagenomic data using HUMAnN3 (v3.0.1) (Abubucker et al., 2012).

### 2.4 Detection of structural variations

Following quality control, SVs were detected from metagenomic sequencing data using SGV-Finder (Zeevi et al., 2019). The SV detection workflow comprised three steps: (i) read mapping and assignment, in which quality-controlled reads were mapped to a reference genome database using the GEM mapper and ambiguously mapped reads were resolved using the Iterative Coverage-based Read Assignment (ICRA) algorithm; (ii) coverage calculation, in which, for each microbial genome in each sample, read coverage was calculated for each genomic bin based on the ICRA-corrected assignments; (iii) population-level SV calling, in which dSVs and vSVs were identified from the per-bin coverage matrix across all samples based on predefined statistical thresholds, followed by clustering of adjacent bins. SGV-Finder detects two types of SVs from metagenomic data: deletion SVs (dSVs) and variable SVs (vSVs). vSVs are genomic segments with a deletion rate of <25% across the population, and their abundance is represented by normalized coverage. dSVs are segments with a deletion rate between 25% and 75% and are represented in a binary manner as presence or absence. We used the reference database provided by SGV-Finder, which is based on the proGenomes database (Mende et al., 2017).

Unless otherwise stated, all parameters were set to the default values recommended in the original publication and associated documentation (Zeevi et al., 2019). It should be noted that for the smaller cohort of 25 multi-site mother-infant longitudinal pairs, sequencing depth was substantially lower and more heterogeneous across ecological niches. Because x_coverage is a user-adjustable parameter in SGV-Finder that affects bin size and coverage-based sample retention, lowering this parameter allowed us to retain more analyzable samples in the low-depth multi-site cohort, albeit with lower analytical stringency and resolution. We therefore used a relaxed x_coverage threshold (0.00009) in the primary multi-site analysis. To evaluate whether the inferred maternal-site contribution pattern was qualitatively stable, we performed a sensitivity analysis using an alternative, more stringent setting (0.0005). The default SGV-Finder setting was not used as the primary comparator because it retained too few samples with detectable SVs in this low-depth multi-site cohort, limiting meaningful cross-site comparison. To improve transparency and reproducibility, a detailed workflow summarizing the SV-calling pipeline, key processing steps, parameter settings, and downstream analyses is shown in **Supplementary Figure 1**.

### 2.5 Quantification and statistical analysis

All statistical analyses were performed in R (v4.1.2). Bray-Curtis distances based on species abundance profiles were calculated using the vegdist function in the vegan package (v2.6.4), and principal coordinates analysis (PCoA) was performed using the ape package (v5.7.1). Group differences between mothers and infants across time points were assessed using PERMANOVA in the vegan package with default settings. To compare microbial diversity patterns between mothers and infants at different time points, pairwise mother-infant distances were extracted from the distance matrix and visualized as boxplots using the ggboxplot function in ggpubr (v0.6.0). Statistical significance among multiple groups was evaluated using the Kruskal-Wallis test. Functional pathway abundance was compared between infant time points using STAMP with Welch’s t-test. Bonferroni correction was applied for multiple testing, and pathways with adjusted P values > 0.05 were considered not significantly different between groups.

Canberra distances were calculated using the vegdist function in the vegan package (v2.6.4) based on the merged SV profiles, and an SV distance matrix was generated. Pairwise SV distances for matched mother-infant pairs and unrelated pairs were then extracted and visualized to assess whether mother-infant pairs showed greater SV similarity than unrelated pairs. Boxplots were generated using ggplot2 (v3.4.4) to visualize SV distances at different time points for each strain. For the functional annotation of SV-associated regions, reference genomes were obtained from the proGenomes database (Mende et al., 2017). Gene sets and their associated metabolic pathway information for each bacterial strain were first retrieved from the proGenomes database. These pathway identifiers were then mapped to specific functional annotations using the KEGG database. In analyses of delivery mode and feeding regimen, tSVs were first identified within each mother-infant pair, and functional pathways were annotated based on tSV-associated regions. Heatmaps were generated using the pheatmap package (version 1.0.12), with log-transformation applied to the pathway occurrence data to facilitate clearer visualization of differences.

## 3 Results

### 3.1 Species composition and functional composition of maternal and infant intestinal microbiomes

**Figure 1:**
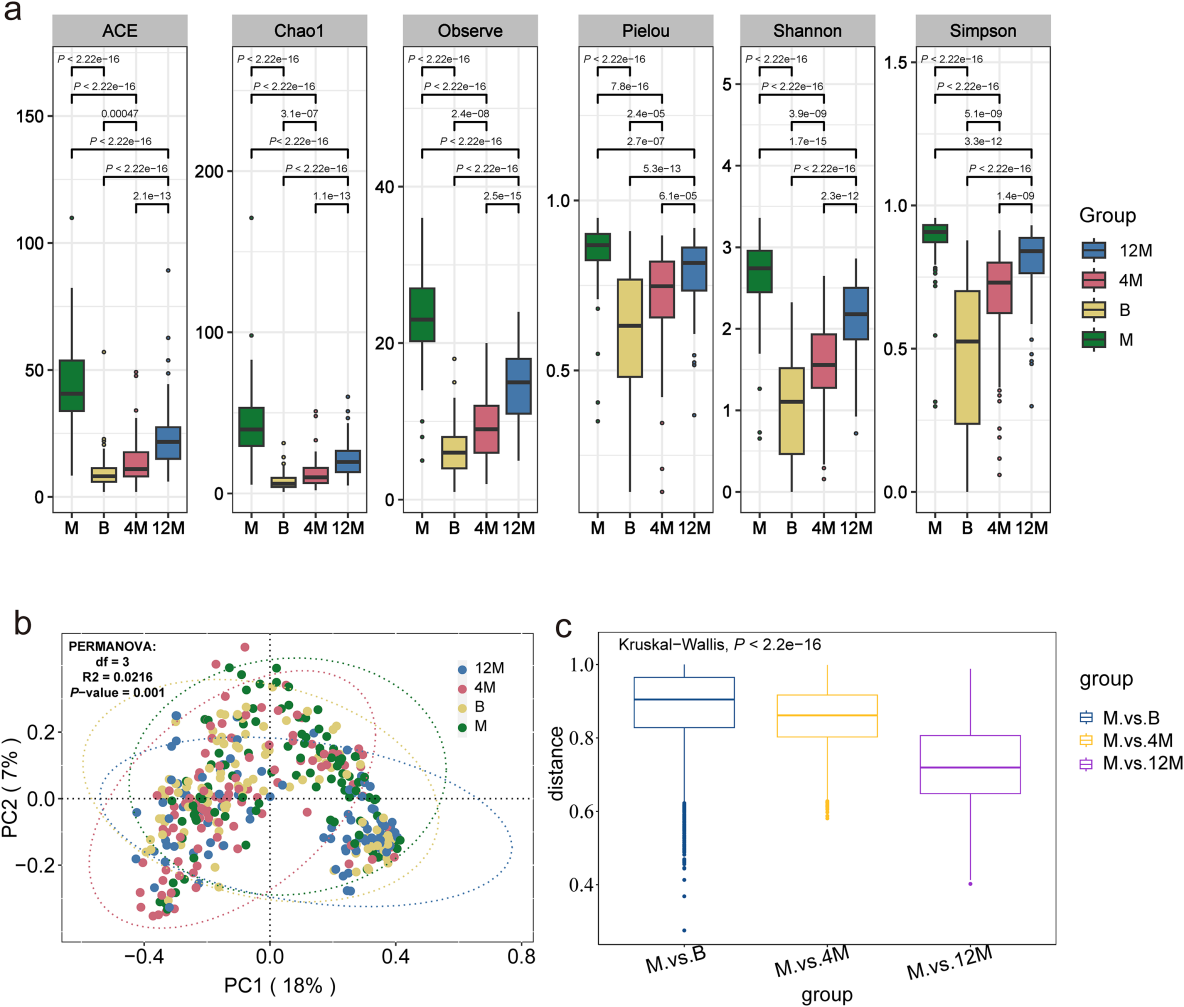
Gut microbiota diversity and community similarity between mothers and infants across different time points. **(a)** Alpha diversity of the gut microbiota in mothers and infants at different sampling time points, reflecting changes in microbial richness and evenness during early-life development. **(b)** Principal coordinates analysis (PCoA) based on Bray-Curtis dissimilarity, illustrating differences in overall gut microbial community composition between mothers and infants at different time points. **(c)** Boxplots of Bray-Curtis distances between mothers and infants at different time points. M, mother; B, infant at birth; 4M, infant at 4 months; 12M, infant at 12 months.

We first characterized the overall development of the gut microbiota in mothers and infants in this longitudinal cohort of 98 mother-infant pairs. Consistent with previous studies, infant gut microbiota diversity increased with age, and alpha diversity as well as community composition differed between mothers and infants (**Figures 1a, b**, *R²* = 0.0216, *P* = 0.001). The results indicated that the infant gut microbiome gradually converged toward the maternal microbiome over time (**Figure 1c**).

At the functional level, age-associated shifts were also observed during early life. Compared with infants at 4 months, newborns showed differences in a limited number of metabolic pathways, primarily related to metabolism and amino acid biosynthesis (**Supplementary Figure 2a**). Functional differentiation became more pronounced between 4 and 12 months, with increasing pathway complexity, including pathways related to carbohydrate utilization such as the phosphotransferase system (PTS) (**Supplementary Figure 2b**). Overall, these results describe the expected maturation of the infant gut microbiome and provide context for the subsequent SV-based analyses.

### 3.2 Maternal and infant intestinal microbiome SV profiling

**Figure 2:**
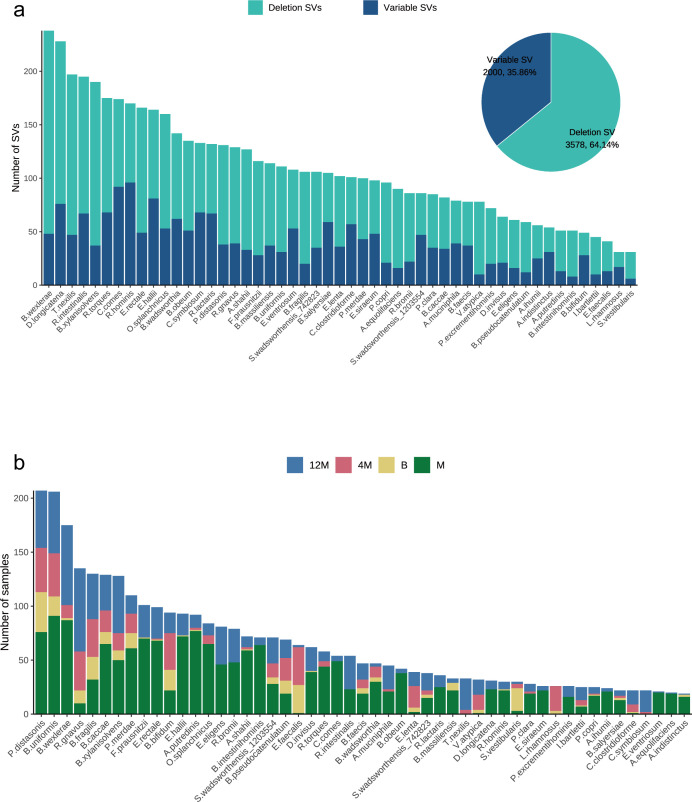
Basic characteristics of structural variants (SVs) detected in the maternal-infant cohort. **(a)** Distribution of different types of structural variants across bacterial strains, illustrating the diversity of SV types identified in the gut microbiome. **(b)** Number of samples in which SVs were detected at each sampling time point for mothers and infants, reflecting temporal differences in SV occurrence across early-life development.

To elucidate the characteristics of SVs within the gut microbiota of mothers and infants, we performed a statistical analysis of the two distinct types of SVs identified in the dataset derived from 98 mother-infant longitudinal cohort. We detected a total of 5,578 SVs in the genomes of 51 reference strains, including 2,000 vSVs and 3,578 dSVs, with 31-238 SVs in each strain (**Figure 2a**). dSVs accounted for a relatively large proportion of all SVs (64%). The strains with the highest numbers of SVs included *Blautia wexlerae* DSM 19850, *Dorea longicatena* AGR2136, and *Tyzzerella nexilis* DSM 1787. In addition, the number of SVs detected per strain was positively correlated with genome size (*R_s_* = 0.436, *P* = 0.00139), indicating that strains with larger genomes tend to harbor more SVs (**Supplementary Figure 3**). This observation is descriptive and likely reflects general genomic properties rather than strain-specific or phylogeny-resolved effects. SVs were detected in infant gut microbiota at all three time points: birth, 4 months, and 12 months. By 12 months, a greater number of strains and samples contained detectable SVs (**Figure 2b**). SV information for each microorganism, along with the full strain names, is provided in **Supplementary Table 1**.

### 3.3 SV-based patterns of mother-infant vertical transmission in the gut microbiome

To investigate whether SVs are associated with vertical transmission-related patterns between mother and infant, we first compared the structural variation similarity between mother-infant pairs based on the Canberra distance of bacterial SV profiles. Not all strains with SVs showed statistically significant differences between mother-infant pairs and unrelated pairs (**Supplementary Figure 4a**). Overall, SV-based genetic differences were smaller in mother-infant pairs than in unrelated pairs, indicating greater SV similarity within mother-infant pairs. Moreover, we observed that the vertical transmission stability of SVs varied across strains. Only SVs from certain strains, such as *Sutterella wadsworthensis* 2_1_59BFAA, *Parabacteroides merdae* ATCC 43184 and *Bacteroides uniformis* ATCC 8492, were observed to be shared at birth and stably maintained in infants (**Supplementary Figure 4b**).

**Figure 3:**
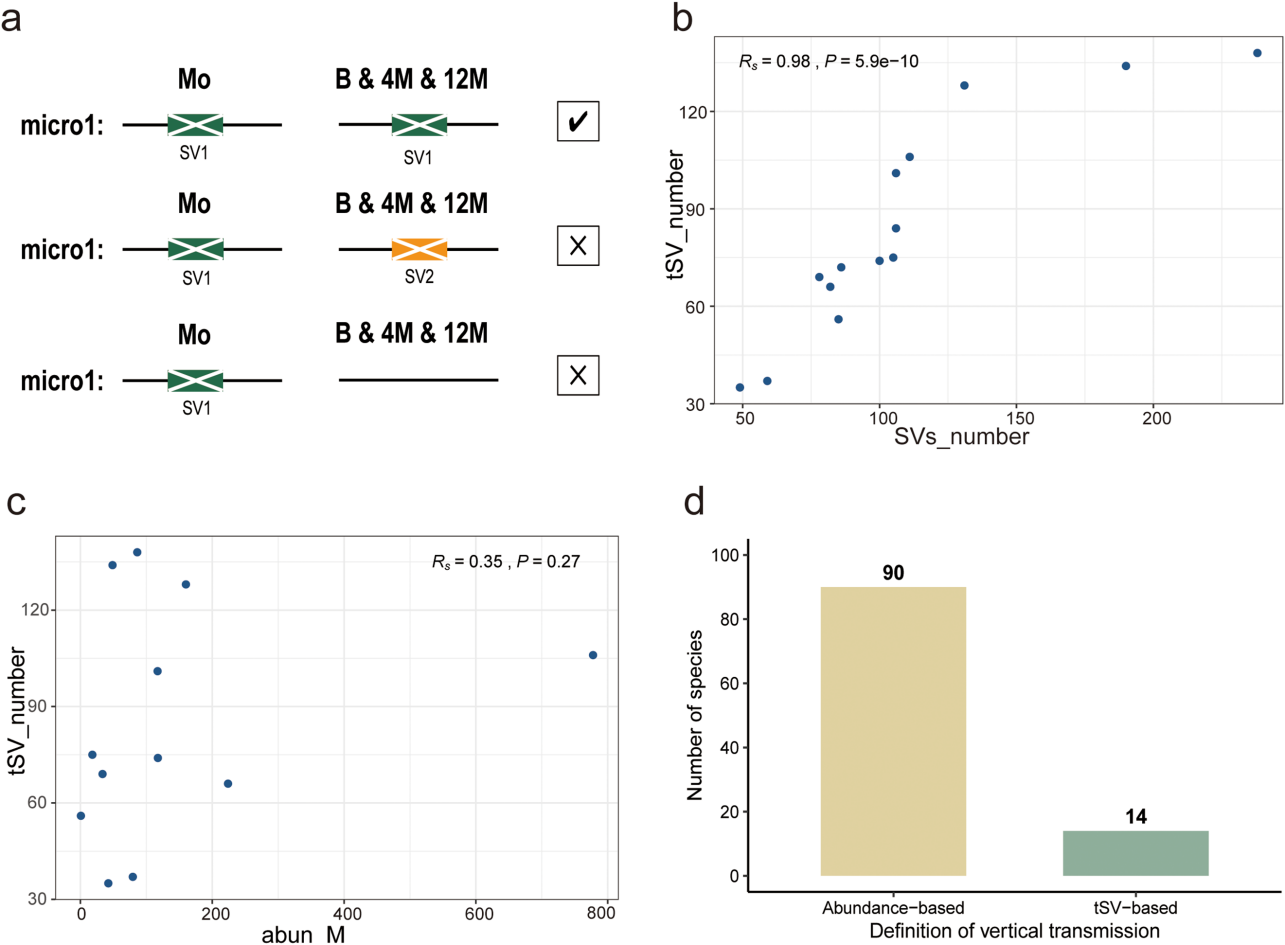
Definition of transmitted structural variants (tSVs) and factors associated with their distribution. **(a)** Schematic overview of tSVs, defined as structural variants (SVs) shared between the mother and infant at birth and retained across all infant sampling time points during the first year of life. **(b)** Correlation between the total number of SVs and the number of tSVs across bacterial strains. **(c)** Correlation between bacterial strain abundance in the mother and the number of tSVs detected in the corresponding mother-infant pairs. abun_M denotes the relative abundance of the bacterial strain in the maternal gut microbiome. **(d)** Comparison of abundance-based species-level sharing and tSV-supported strain-level patterns, highlighting the more stringent criteria imposed by SV-based analysis. *R_s_* denotes Spearman’s rank correlation coefficients, and *P* values indicate the statistical significance of the corresponding correlation tests.

To further investigate SVs potentially involved in vertical transmission, we defined a set of transmitted SVs (tSVs) on a per mother-infant pair basis as SVs that were detected in both the mother and the infant at birth and that remained present in the infant at all subsequent sampling time points (4 months and 12 months) (**Figure 3a**). This definition captures SVs that are both initially shared at birth (early sharing) and longitudinally maintained (persistence). A total of 671 transmitted dSVs (tdSVs) were detected, among which *Bacteroides xylanisolvens* XB1A contained the most SVs of this type; 504 transmitted vSVs (tvSVs) were detected, among which *Bacteroides salyersiae* contained the most. We found that the number of tSVs of each bacterial strain was positively correlated with the total number of SVs of the corresponding strain (*R_s_* = 0.98, *P* = 5.9e-10) (**Figure 3b**), but the number of tSVs of each bacterial strain was not related to the abundance of the corresponding bacteria in the mother (**Figure 3c**). While abundance-based analysis identified 90 bacterial species shared between mothers and infants across all sampled time points (birth, 4 months, and 12 months), our pair-level tracking of tSVs further restricted this set to 14 bacterial strains showing tSV-supported strain-level patterns related to vertical transmission (**Figure 3d**, **Supplementary Table 2**). Accordingly, to distinguish birth-time SV-supported strain-level patterns from persistent tSV-supported patterns, we separately summarized bacterial strains inferred to be shared between mothers and infants based on SVs detected in both the mother and the infant at birth, and included this category in **Supplementary Table 2**.

### 3.4 Functions of SVs

Microbial SV regions may contain functional genes that influence host-microbe interactions. The presence of SVs therefore represents a potential mechanism for functional diversification, underscoring the importance of investigating the genes associated with SVs. However, it is important to note that all functional interpretations in this study are based on bioinformatic annotation rather than direct experimental validation.

**Figure 4:**
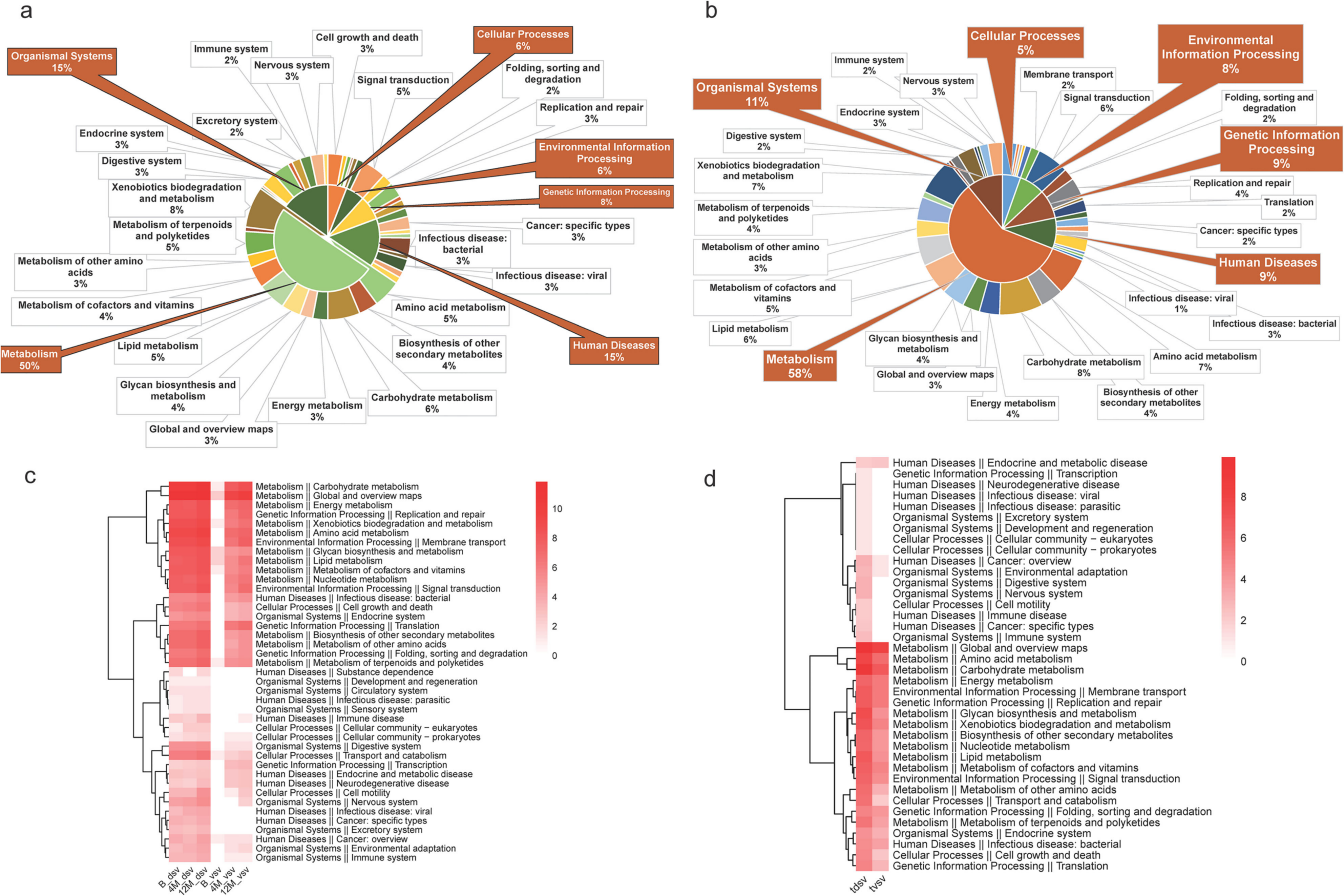
Functional profiles of structural variants (SVs) and transmitted structural variants (tSVs) in infants across three time points. **(a)** Pie chart summarizing the functional composition of all SVs detected in infant samples, based on KEGG pathway annotations. **(b)** Pie chart showing the functional composition of all tSVs identified in the cohort. **(c)** Heatmap comparing the functional profiles of deletion SVs (dSVs) and variable SVs (vSVs) across the three infant sampling time points (birth, 4 months, and 12 months). **(d)** Heatmap illustrating the functional enrichment patterns of transmitted deletion SVs (tdSVs) and transmitted variable SVs (tvSVs). For all analyses, KEGG functional pathways were annotated and summarized at the first- and second-level classifications, and heatmaps display pathways aggregated at the secondary classification level.

For infants, SV-associated regions were mostly annotated to metabolism-related pathways, such as carbohydrate, lipid, and amino acid metabolism pathways (**Figure 4a**). The functional proportions of SVs differed across each stage of infancy (**Figure 4c**). Overall, dSVs and vSVs showed largely overlapping functional annotations. For the same SV type, the enriched functional pathways at different stages were not consistent. For most pathways, enrichment increased at 12 months, which may be related to the increase in the number of SVs with age.

The presence of tSVs suggests that specific genomic variants potentially involved in vertical transmission, shared between mothers and infants and maintained over time, may be associated with stable microbial functional profiles in the infant. Given that infancy is a critical period for the development of various systems, it is essential to analyze the functions associated with tSVs (**Figures 4b, d**). For tSVs, tdSVs and tvSVs displayed broadly similar functional profiles in infant feces. Most of the pathways enriched by tdSVs and tvSVs are metabolic pathways, such as carbohydrate metabolism, amino acid metabolism, energy metabolism, lipid metabolism, and glycan biosynthesis and metabolism. Pathways related to the type IV secretion system (T4SS) module, such as those involved in transmembrane transport and signal transduction, were also enriched. Collectively, these functional annotations suggest that tSV-associated regions may harbor genes involved in key metabolic and adaptive processes, although these inferences remain annotation-based.

### 3.5 Influences of mode of delivery and feeding regimen on the infant gut microbiota

**Figure 5:**
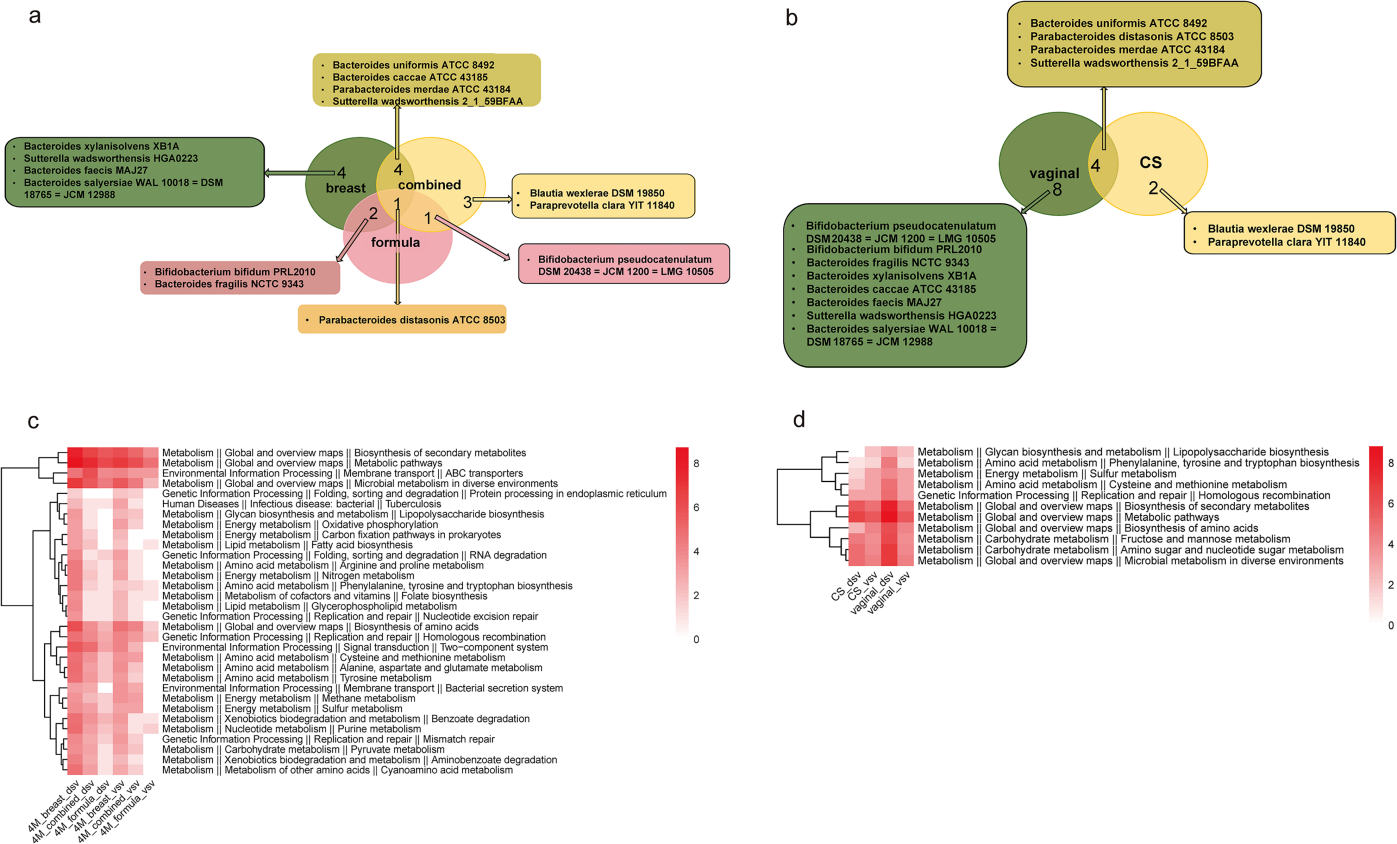
Functions of vertically transmitted strains and transmitted structural variants (tSVs) under different feeding regimens and delivery modes. **(a)** Bacterial strains inferred to be vertically transmitted under three feeding regimens: Breast (n = 66), Combined (n = 19), and Formula (n = 11). **(b)** Bacterial strains inferred to be vertically transmitted under two delivery modes: CS (n = 15) and Vaginal (n = 83). **(c)** Differential tSV-associated functional pathways among the three feeding regimens. **(d)** Differential tSV-associated functional pathways between the two delivery modes. Breast, Breastfeeding; Combined, Mixed feeding; Formula, Formula feeding; CS, Cesarean section; Vaginal, Vaginal delivery.

We evaluated the associations of delivery mode and feeding regimen with SV-based patterns related to vertical transmission between mother and infant. Given the subgroup-based comparisons and unequal subgroup sizes, these analyses were interpreted as exploratory and descriptive rather than confirmatory. Differences in the patterns of shared microbiota and tSVs were observed across feeding-regimen groups. We grouped mother-infant pairs according to infant feeding patterns at 4 months of age. For feeding regimen, the cohort was divided into breastfeeding (n = 66), mixed feeding (n = 19), and formula feeding (n = 11) groups. Using the presence of tSVs as an indicator, we identified tSV-supported strain-level sharing patterns in gut microbiota. The results suggested that breastfeeding was associated with a higher level of tSV-supported strain-level patterns related to vertical transmission between mother and infant, followed by mixed feeding, and then formula feeding (**Figure 5a**). *Parabacteroides distasonis* ATCC 8503 was detected among tSV-supported taxa identified across all three feeding groups. Functional annotation of tSV-associated regions in these taxa suggested that different feeding methods may affect pathways related to metabolism, bacterial infection, and the endocrine system (**Figure 5c**). Compared with the other feeding groups, the breastfed group descriptively showed a broader set of tSV-associated pathways related to energy, lipid, amino acid, cofactor, and vitamin metabolism, whereas the formula-fed group showed fewer such pathway occurrences.

tSV-supported patterns related to vertical transmission differed between delivery-mode groups. Compared with the cesarean section group (CS, n = 15), the vaginal-delivery group (n = 83) showed a higher number of tSV-supported strains per mother-infant pair. Specifically, tSV-based analyses supported the potential vertical transmission of 12 bacterial strains in the vaginal delivery group, compared to 6 in the cesarean section group (**Figure 5b**). *Bacteroides uniformis* ATCC 8492, *Parabacteroides distasonis* ATCC 8503, *Parabacteroides merdae* ATCC 43184, and *Sutterella wadsworthensis* 2_1_59BFAA were common to both groups. Our data indicate that *Bacteroides* species were more frequently represented among the taxa showing tSV-supported patterns related to vertical transmission in the vaginal-delivery group. Different delivery-mode groups showed differences in tSV-associated pathways, many of which were related to metabolic functions (**Figure 5d**). The vaginal delivery group descriptively showed more pathway occurrences related to energy metabolism, amino acid metabolism, fructose and mannitol metabolism, and lipopolysaccharide biosynthesis than the cesarean section group.

### 3.6 SV-based microbial patterns related to vertical transmission across maternal and infant ecological niches

**Figure 6:**
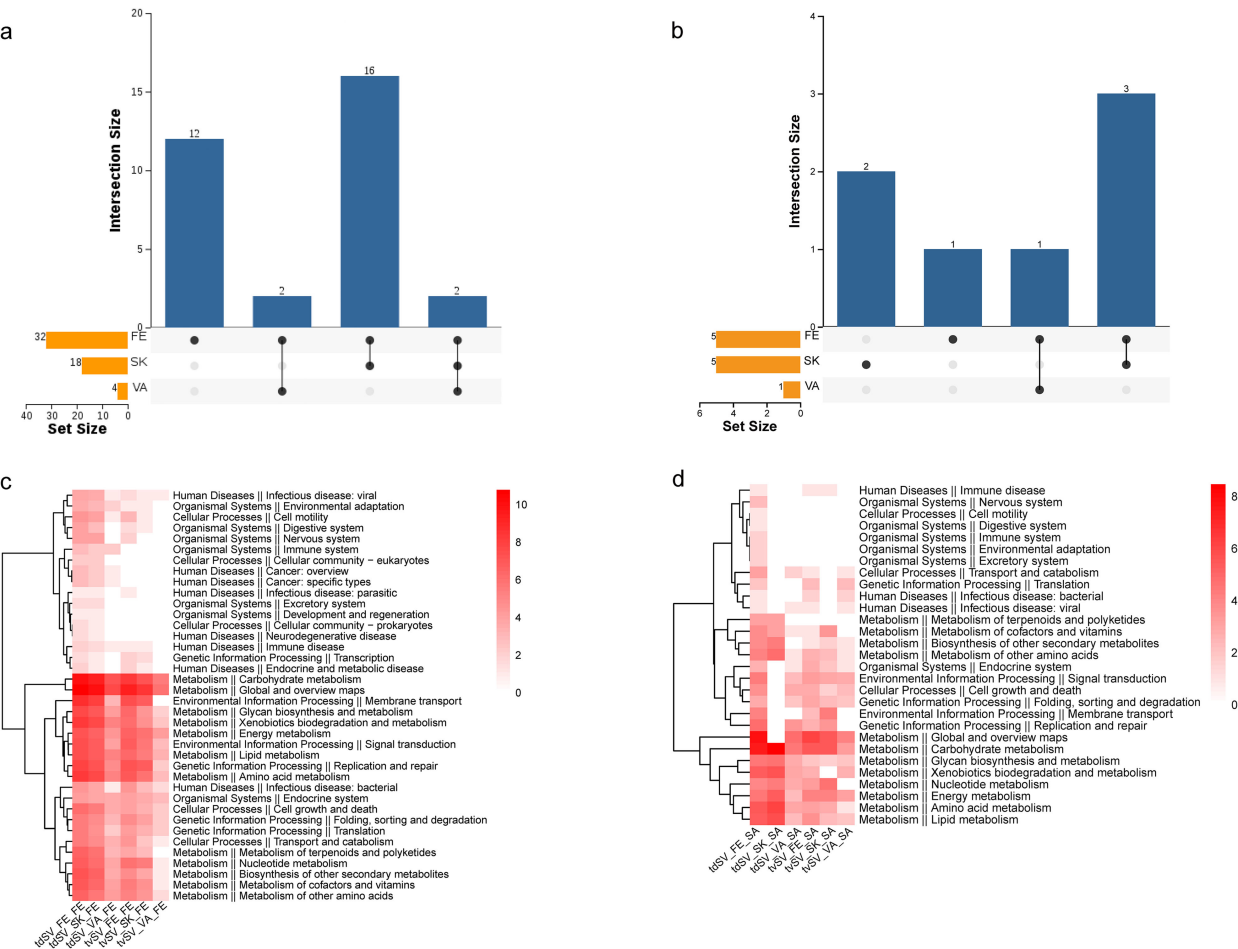
Vertical transmission of microorganisms between different ecological niches of mother and infant. Bacterial strains inferred to be vertically transmitted from different maternal ecological niches to the infant gut and oral cavity **(a, b)**, with corresponding tSV functional enrichment heatmaps **(c, d)**. Functional annotations were performed using KEGG pathways and summarized at the secondary classification level to facilitate comparison of functional profiles across niches. In the UpSet plots, “Intersection Size” indicates the number of elements shared among specific set combinations, whereas “Set Size” represents the total number of elements in each individual set. FE, feces; SK, skin; VA, vagina; SA, saliva.

To explore SV-based patterns potentially related to vertical transmission across maternal and infant ecological niches, the metagenomic data from 25 mother-infant pairs sampled across different ecological niches were analyzed. We analyzed samples from various ecological niches of mothers and infants within the first three days after birth to identify the bacteria and SVs showing inferred vertical transmission from mothers to newborns at birth. First, we examined bacteria inferred to be involved in vertical transmission from different maternal ecological niches to the newborn gut. The composition of these putatively transmitted bacteria differed across maternal ecological niches in the newborn intestine (**Supplementary Table 3**). For the neonatal gut microbiome, the maternal gut showed the strongest inferred signal of shared SV-supported strains with the infant gut, while smaller signals were also observed for maternal skin and vaginal sites. Maternal oral sites showed the weakest inferred contribution to the neonatal gut microbiome in our analysis, as no robust evidence of strain sharing between mother and infant was detected (**Figure 6a**). To assess the robustness of this pattern, sensitivity analysis across two alternative x_coverage settings (0.00009 and 0.0005) showed that, although the absolute number of inferred transmitted strains varied, the overall maternal-site contribution pattern remained qualitatively similar (**Supplementary Figure 5**). In addition, the functions of tSVs also differed across ecological niches. Functions related to human diseases and biological systems were more frequently represented among SVs shared between maternal and neonatal gut samples (**Figure 6c**). Across the three ecological niches, intestine, skin, and vagina, most annotated pathways were related to metabolism and genetic information processing.

For bacteria inferred to be vertically transmitted from different maternal ecological niches to the newborn oral cavity (**Supplementary Table 4**), the maternal gut emerged as the predominant inferred contributor to the infant oral microbiome under our analytical framework, followed by the skin, whereas the vagina showed the smallest inferred contribution (**Figure 6b**). SVs in functional pathways related to the immune system, digestive system, and environmental adaptation were predominantly associated with inferred vertical transmission from the maternal intestinal microbiota to the newborn oral cavity (**Figure 6d**).

## 4 Discussion

In this study, we analyzed two European mother-infant cohorts to explore patterns of maternal contribution to infant microbiome assembly from the perspective of microbial SVs. We examined potential associations of delivery mode and infant-feeding practices with the transmission of microbes and their SVs, and further explored SV-related transmission patterns across different ecological niches. Shared SVs within mother-infant pairs provide complementary support for strain-level vertical transmission.

We found that SVs may be involved in vertical transmission-related patterns between mother and infant, with each mother-infant pair exhibiting specific SV transmission patterns. These differences may reflect distinct ecological contexts and selection pressures. Such pressures may favor the transmission of bacteria carrying particular SV profiles in specific ecological contexts (Zeevi et al., 2019). When analyzing SVs within individual strains, we found that not all SVs met our criteria for apparent transmission and persistence in infants. Only a small fraction of SVs in stable strains met our tSV criteria and remained detectable across infant time points. This finding aligns with reports in adults showing variable within-individual stability across microbial SVs, with only a subset exhibiting high stability (Chen et al., 2021). Furthermore, we observed a positive correlation between genome size and the number of structural variants across strains, suggesting that general genomic properties may contribute to the overall distribution of SVs. Future studies incorporating phylogeny-aware comparative frameworks will be important to disentangle the relative contributions of genome size, evolutionary history, and ecological factors to the accumulation of structural variation across bacterial lineages.

SVs are large-scale genomic alterations with potential functional consequences. Shared SVs within mother-infant pairs provide complementary support for strain-level vertical transmission. Among the 98 mother-infant pairs analyzed, pair-level tracking of tSVs identified 14 bacterial strains potentially associated with vertical transmission. This observation suggests the stable maintenance of shared genomic features. Such persistence may be linked to the functional potential of these microbes within the gut environment. The majority of the bacterial strains that showed patterns consistent with potential mother-infant vertical transmission in our analysis have been associated with host health outcomes in prior studies, such as *Bacteroides fragilis* (Chan et al., 2019), *Bacteroides faecis* (Mohebali et al., 2023), *Parabacteroides distasonis* (Wang et al., 2019), and *Bifidobacterium bifidum* PRL2010 (Turroni et al., 2014; Duranti et al., 2018). These strains may be important during early-life community assembly.

Functional annotation of SV-related genes provided insights into the potential functional implications of SV-associated regions. Prior work in adults reported functional enrichment of SV-associated genes in ABC-2 and type-IV secretion system (Zeevi et al., 2019). In our infant cohort, SV-associated functions were more frequently annotated to metabolic pathways. This contrast suggests that SV-linked functional annotations may differ across life stages. We further observed that the tSVs were enriched in pathways associated with T4SS modules, which have been implicated in microbial adaptation to environmental changes (Zeevi et al., 2019). These findings suggest that SV-associated genomic regions may encode functions related to microbial adaptation and persistence. Although these interpretations are based on bioinformatic annotation rather than direct experimental validation, the functional annotation of SV-related genes provides a preliminary basis for inferring the potential relevance of SV-associated regions. Future studies integrating molecular experiments and functional validation will be needed to clarify the physiological significance of these SV-associated features.

Across feeding methods, our SV-based analysis showed a descriptive descending pattern in apparent strain sharing in the breastfeeding, mixed feeding, and formula feeding groups. Analysis of SV-associated pathways across feeding modes identified differential enrichment in the breastfed group. These pathways were more often annotated to utilization of breast milk-related nutrients and vitamin-associated functions. They were also associated with microbial maintenance-related modules. These findings are consistent with the idea that breast milk provides distinct nutritional substrates and ecological conditions for the infant microbiota (Boudry et al., 2021). Our data further suggested that, compared with the mode of delivery, different feeding regimens are associated with the vertical transmission of a greater variety of SVs with diverse functions between mother and infant. We also observed that both vaginal delivery and breastfeeding were associated with transmission of taxa reported to be associated with host health outcomes in prior studies. For instance, *Bacteroides fragilis* has been associated with reduced intestinal inflammation, and *Sutterella wadsworthensis* has been reported in relation to cardiovascular disease (Jiang et al., 2017; Cuadrat et al., 2023). However, because these analyses rely on subgroup comparisons with unequal sample sizes and a descriptive statistical framework, they should be interpreted as exploratory and hypothesis-generating rather than as definitive evidence that feeding regimen or delivery mode causally determines SV-supported transmission patterns.

Our analysis of strain sharing, inferred from SVs, suggests that bacteria from the mother’s intestines, vagina, and skin may contribute to the establishment of the infant gut and oral microbiomes during early life. The estimated contribution of the vagina and skin is smaller than that of the intestine. This may reflect differences in local ecological conditions across body sites. The relatively similar ecological conditions of the intestine and oral cavity may facilitate colonization by certain taxa. We speculate that *Bacteroides uniformis* ATCC 8492, which is inferred to show vertical transmission-related patterns from the mother’s intestines and vagina to the newborn’s intestines, may originate from the mother’s intestines, as this bacterium has been used as a marker to distinguish vaginal and fecal sources (Zou et al., 2016). Consequently, the analysis of bacterial microbiota sources should take into account the ecological niches where these bacterial species are actually colonized. The majority of bacterial strains in the maternal vagina that may be vertically transmitted to the newborn overlapped with those transmitted from the maternal intestine. This suggests that newborns born vaginally are mainly exposed to bacteria from the mother’s intestines, with fewer bacteria vertically transmitted from the mother’s vagina, possibly owing to niche-specific selective constraints that limit the persistence or transfer of vaginal taxa outside the vaginal environment (Kwon and Lee, 2022).

Several limitations should be noted. First, postnatal co-habitation and shared diet can drive microbiome convergence. These household factors can also drive strong horizontal transmission. Therefore, our analysis cannot fully separate perinatal vertical transmission from postnatal household transfer. Longitudinal sampling (birth, 4 months, and 12 months) helps contextualize early sharing, but residual confounding may remain. Second, the absence of orthogonal validation, such as SNP-based strain tracking, limits our ability to definitively resolve transmission dynamics. Core-genome SNP analysis on the same cohort would provide the gold standard for validating SV-based transmission inferences. SVs provide complementary evidence for vertical transmission and clues regarding functional alterations in microbial strains. Therefore, we consider integrating SNP- and SV-based approaches to be an important direction for future research. Third, our SV analysis focused on dSVs and vSVs. Other SV classes were not assessed. Therefore, our analysis may underestimate SV diversity and may miss strain differences driven by SV classes not captured by the current reference-anchored framework. Fourth, another limitation is the unequal subgroup sizes, particularly for delivery mode, which may have affected the stability of subgroup comparisons. Finally, our approach is reference-anchored and depends on the completeness and representativeness of the strain catalog. Some SVs may also be dynamic or reversible under selection (Belikova et al., 2020). Future work should integrate SV and SNP layers, expand catalogs via long-read or *de novo* assemblies, and adopt designs that better separate vertical inheritance from household-driven horizontal transmission and shared environmental effects. Such designs could include richer household metadata, multiple siblings, or alternative caregiving contexts.

In conclusion, this study provides complementary insights into mother-to-infant microbial transmission and offers a useful direction for future research, and may inform future investigations into health outcomes in mother-infant dyads.

## Data Availability

The original contributions presented in the study are included in the article/**Supplementary Material**. Further inquiries can be directed to the corresponding authors.
