# Supplementary material for: Structural variation analysis suggests strain-level maternal-infant microbial transmission in early life
Source: Front Cell Infect Microbiol. 2026 May 19;16:1765801. doi: 10.3389/fcimb.2026.1765801 (PMC13226579; doi:10.3389/fcimb.2026.1765801)
Supplement: Supplementary file 1 [file Table1.docx]

Supplementary Material

# Supplementary Figures and Tables

## Supplementary Figures

**
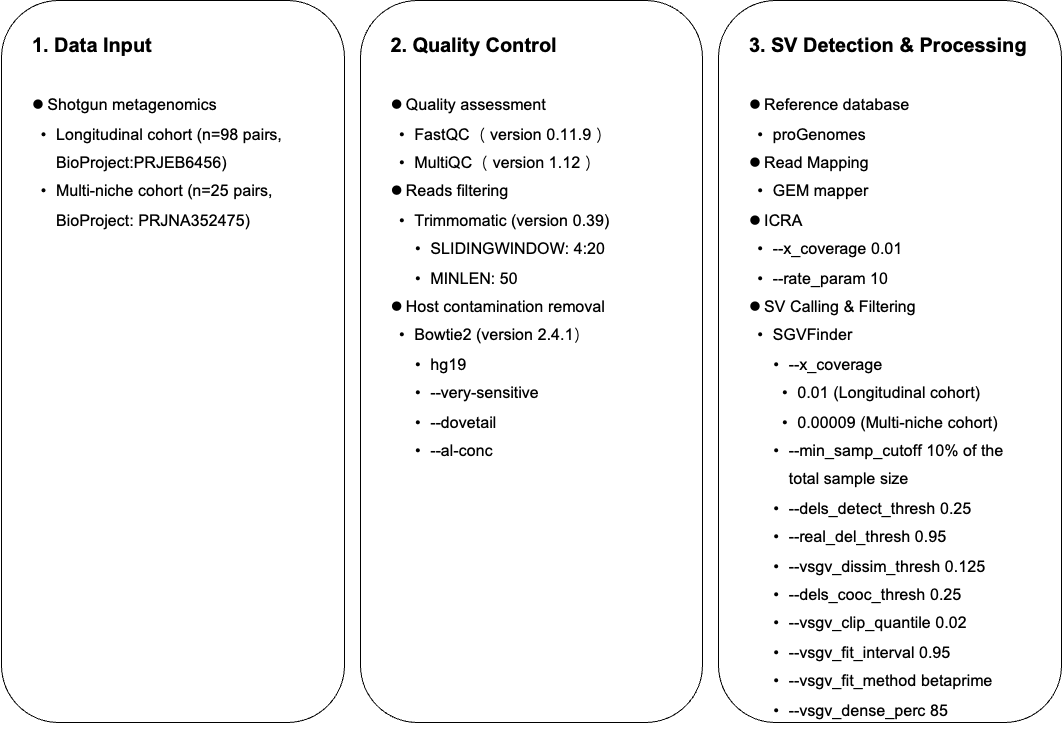
**

**Supplementary Figure 1. Detailed workflow of SV-based analysis**

**
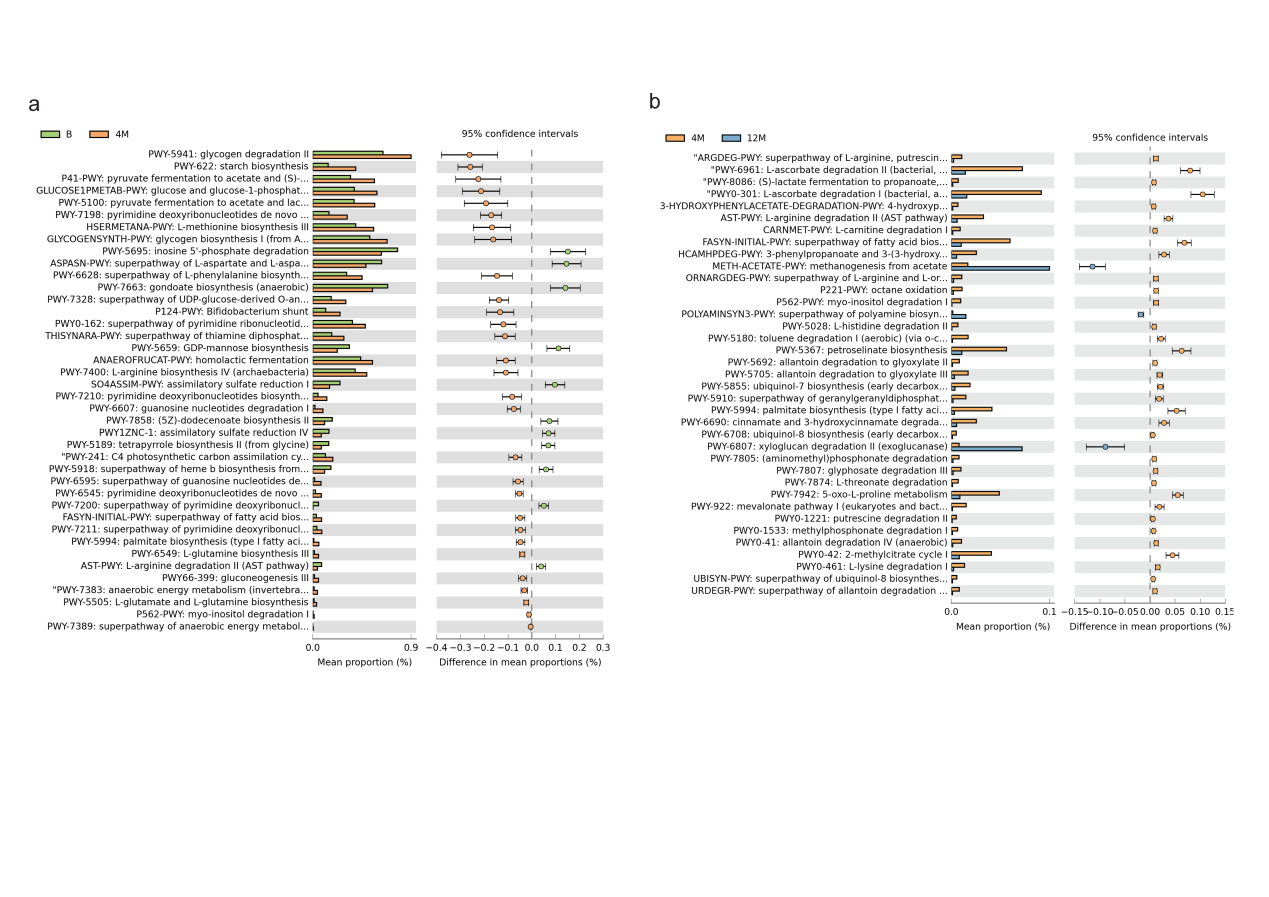
**

**Supplementary Figure 2. Different functional pathways of infants at different time points.** (a) Different functional pathways of infants at birth and 4 months old. (b) Different functional pathways of infants at 4 months old and 12 months old (Ratio of proportions = 5).


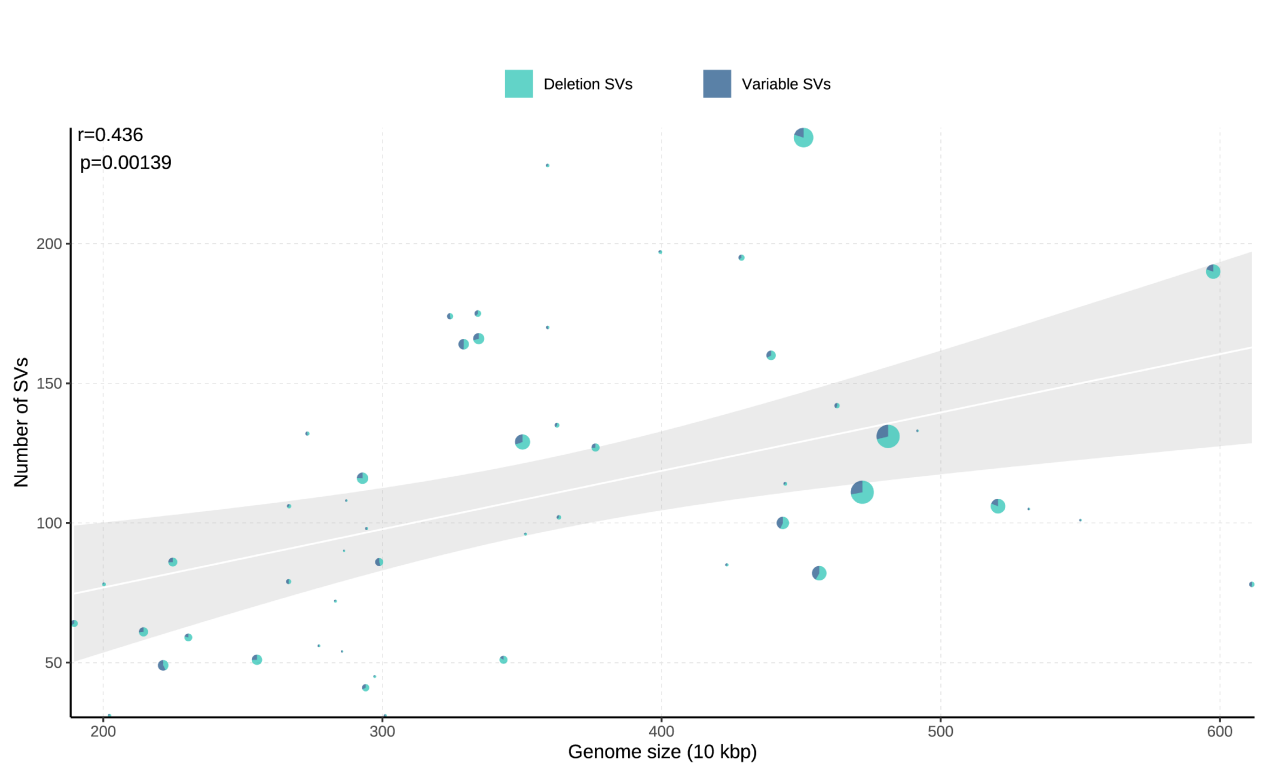


**Supplementary Figure 3. Scatter plot of bacterial species genome size and SV number.** Correlation analysis between the genome size of each bacterial species and its SV number. Each point in the scatter plot represents each bacterial species, and the pie chart represents the proportion of dSVs and vSVs contained in each bacterial species.


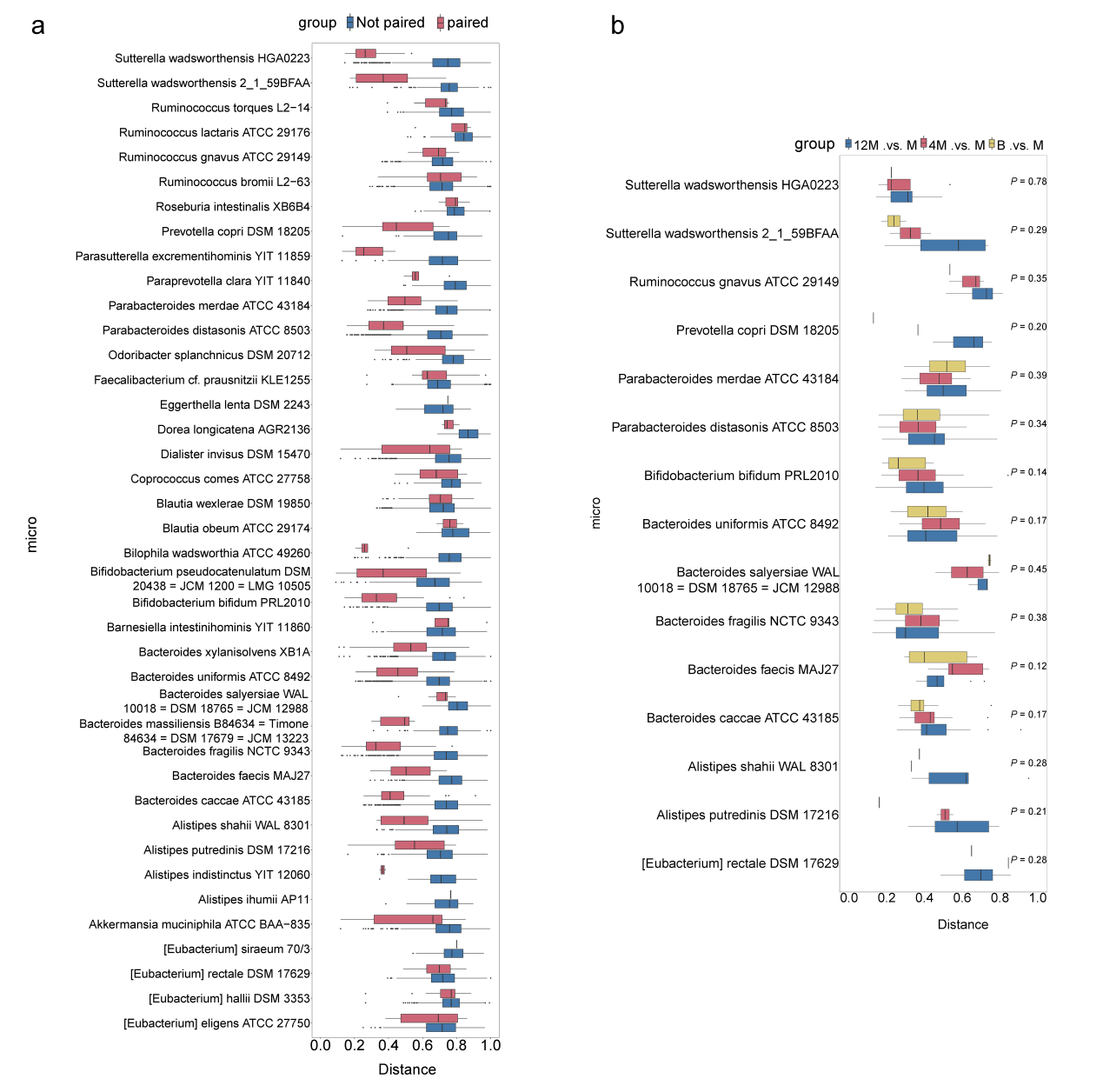


**Supplementary Figure 4. Similarity analysis of intestinal microbial structural variation between mother and infant.** (a) Similarity analysis of microbial structural variation between mother and infant pairs and unrelated pairs. (b) Comparison of similarity of microbial structural variation between mothers and their infants for each species at three time points.


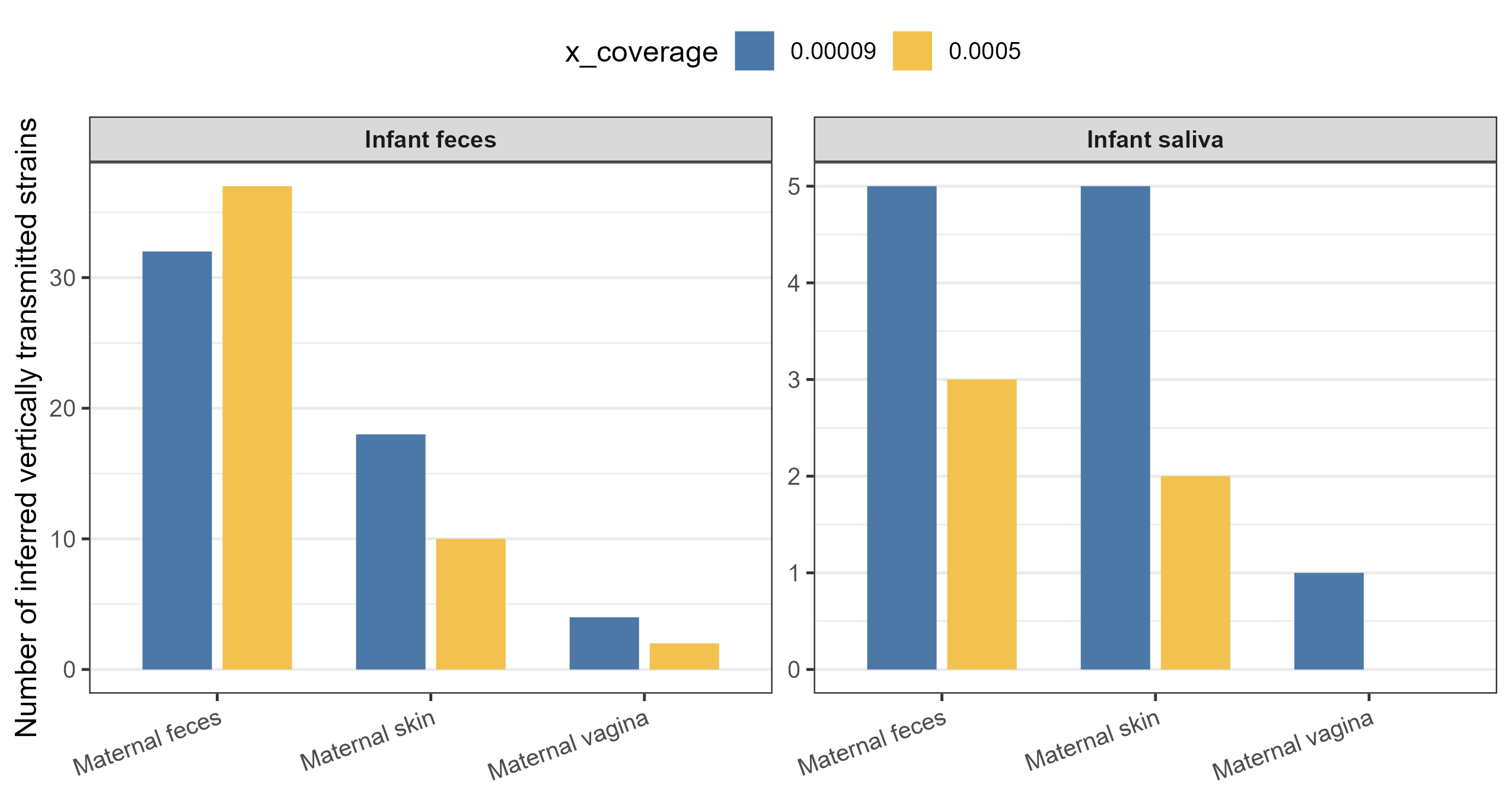


**Supplementary Figure 5. Sensitivity analysis of inferred maternal-site contributions under two alternative x_coverage settings in the 25-pair multi-site cohort.** Bar plots show the number of inferred vertically transmitted strains from maternal fecal, skin, and vaginal microbiomes to infant fecal and saliva microbiomes under two alternative x_coverage settings (0.00009 and 0.0005). Although the absolute counts varied, the overall maternal-site contribution pattern remained qualitatively similar across the tested settings.

## Supplementary Tables

**Supplementary Table 1.** **Summary of microbial species and their associated structural variations (SVs) in the longitudinal mother-infant cohort (n=98 pairs).**

| **Organism name** | **Abbreviation** | **Number of SVs** | **Number of samples** |
| --- | --- | --- | --- |
| Adlercreutzia equolifaciens DSM 19450 | A.equolifaciens | 90 | 20 |
| Alistipes ihumii AP11 | A.ihumii | 56 | 24 |
| Alistipes indistinctus YIT 12060 | A.indistinctus | 54 | 19 |
| Akkermansia muciniphila ATCC BAA-835 | A.muciniphila | 79 | 45 |
| Alistipes putredinis DSM 17216 | A.putredinis | 51 | 92 |
| Alistipes shahii WAL 8301 | A.shahii | 127 | 72 |
| Bifidobacterium bifidum PRL2010 | B.bifidum | 49 | 94 |
| Bacteroides caccae ATCC 43185 | B.caccae | 82 | 129 |
| Bacteroides faecis MAJ27 | B.faecis | 78 | 47 |
| Bacteroides fragilis NCTC 9343 | B.fragilis | 106 | 130 |
| Barnesiella intestinihominis YIT 11860 | B.intestinihominis | 51 | 71 |
| Bacteroides massiliensis B84634 = Timone 84634 = DSM 17679 = JCM 13223 | B.massiliensis | 114 | 33 |
| Blautia obeum ATCC 29174 | B.obeum | 135 | 42 |
| Bifidobacterium pseudocatenulatum DSM 20438 = JCM 1200 = LMG 10505 | B.pseudocatenulatum | 59 | 69 |
| Bacteroides salyersiae WAL 10018 = DSM 18765 = JCM 12988 | B.salyersiae | 105 | 22 |
| Bacteroides uniformis ATCC 8492 | B.uniformis | 111 | 206 |
| Bilophila wadsworthia ATCC 49260 | B.wadsworthia | 142 | 47 |
| Blautia wexlerae DSM 19850 | B.wexlerae | 238 | 175 |
| Bacteroides xylanisolvens XB1A | B.xylanisolvens | 190 | 128 |
| [Clostridium] clostridioforme 2_1_49FAA | C.clostridioforme | 101 | 22 |
| Coprococcus comes ATCC 27758 | C.comes | 174 | 54 |
| [Clostridium] symbiosum WAL-14673 | C.symbiosum | 133 | 22 |
| Dialister invisus DSM 15470 | D.invisus | 64 | 62 |
| Dorea longicatena AGR2136 | D.longicatena | 228 | 31 |
| [Eubacterium] eligens ATCC 27750 | E.eligens | 61 | 81 |
| Enterococcus faecalis ATCC 29212 | E.faecalis | 41 | 64 |
| [Eubacterium] hallii DSM 3353 | E.hallii | 164 | 93 |
| Eggerthella lenta DSM 2243 | E.lenta | 102 | 39 |
| [Eubacterium] rectale DSM 17629 | E.rectale | 166 | 99 |
| [Eubacterium] siraeum 70/3 | E.siraeum | 98 | 26 |
| Eubacterium ventriosum ATCC 27560 | E.ventriosum | 108 | 21 |
| Faecalibacterium cf. prausnitzii KLE1255 | F.prausnitzii | 116 | 101 |
| Intestinibacter bartlettii DSM 16795 | I.bartlettii | 45 | 25 |
| Lactobacillus rhamnosus GG | L.rhamnosus | 31 | 26 |
| Odoribacter splanchnicus DSM 20712 | O.splanchnicus | 160 | 84 |
| Paraprevotella clara YIT 11840 | P.clara | 85 | 28 |
| Prevotella copri DSM 18205 | P.copri | 96 | 25 |
| Parabacteroides distasonis ATCC 8503 | P.distasonis | 131 | 207 |
| Parasutterella excrementihominis YIT 11859 | P.excrementihominis | 72 | 26 |
| Parabacteroides merdae ATCC 43184 | P.merdae | 100 | 110 |
| Ruminococcus bromii L2-63 | R.bromii | 86 | 79 |
| Ruminococcus gnavus ATCC 29149 | R.gnavus | 129 | 135 |
| Roseburia hominis A2-183 | R.hominis | 170 | 30 |
| Roseburia intestinalis XB6B4 | R.intestinalis | 195 | 54 |
| Ruminococcus lactaris ATCC 29176 | R.lactaris | 132 | 36 |
| Ruminococcus torques L2-14 | R.torques | 175 | 58 |
| Streptococcus vestibularis F0396 | S.vestibularis | 31 | 30 |
| Sutterella wadsworthensis 2_1_59BFAA | S.wadsworthensis_742823 | 106 | 38 |
| Sutterella wadsworthensis HGA0223 | S.wadsworthensis_1203554 | 86 | 71 |
| Tyzzerella nexilis DSM 1787 | T.nexilis | 197 | 33 |
| Veillonella atypica KON | V.atypica | 78 | 32 |

**Supplementary Table 2. Comparison of mother-infant microbial sharing at three analytical levels in the longitudinal mother-infant cohort (n = 98 pairs).** This table compares mother-infant microbial sharing at three analytical levels in the longitudinal mother-infant cohort (n = 98 pairs): birth-time SV-supported strain-level patterns, representing strains supported by SV sharing at birth; tSV-supported strain-level patterns, representing strains supported by SVs shared at birth and maintained across subsequent infant time points; and abundance-based species-level patterns, representing species detected in both mother and infant at birth and remaining detectable at subsequent infant time points.

| **Birth-time SV-supported strain-level patterns** | **tSV-supported strain-level patterns** | **Abundance-based species-level patterns** |
| --- | --- | --- |
| Bacteroides fragilis NCTC 9343 | Bacteroides fragilis NCTC 9343 | Bifidobacterium adolescentis |
| Parabacteroides distasonis ATCC 8503 | Bifidobacterium pseudocatenulatum DSM 20438 = JCM 1200 = LMG 10505 | Bifidobacterium longum |
| Bacteroides xylanisolvens XB1A | Bacteroides xylanisolvens XB1A | Bacteroides fragilis |
| Bacteroides uniformis ATCC 8492 | Parabacteroides distasonis ATCC 8503 | Bacteroides ovatus |
| Bifidobacterium bifidum PRL2010 | Bacteroides uniformis ATCC 8492 | Bacteroides uniformis |
| Bacteroides massiliensis B84634 = Timone 84634 = DSM 17679 = JCM 13223 | Bacteroides caccae ATCC 43185 | Bacteroides vulgatus |
| Bacteroides faecis MAJ27 | Parabacteroides merdae ATCC 43184 | Alistipes finegoldii |
| Bacteroides caccae ATCC 43185 | Sutterella wadsworthensis HGA0223 | Eubacterium sp. CAG 180 |
| Bifidobacterium pseudocatenulatum DSM 20438 = JCM 1200 = LMG 10505 | Bacteroides faecis MAJ27 | Roseburia faecis |
| Parabacteroides merdae ATCC 43184 | Bifidobacterium bifidum PRL2010 | Intestinibacter bartlettii |
| Bacteroides salyersiae WAL 10018 = DSM 18765 = JCM 12988 | Bacteroides salyersiae WAL 10018 = DSM 18765 = JCM 12988 | Faecalibacterium prausnitzii |
| Blautia wexlerae DSM 19850 | Blautia wexlerae DSM 19850 | Ruminococcus bicirculans |
| Sutterella wadsworthensis HGA0223 | Sutterella wadsworthensis 2 1 59BFAA | Escherichia coli |
| Alistipes indistinctus YIT 12060 | Paraprevotella clara YIT 11840 | Enterococcus faecalis |
| Sutterella wadsworthensis 2_1_59BFAA |  | Streptococcus parasanguinis |
| Alistipes putredinis DSM 17216 |  | Streptococcus salivarius |
| Faecalibacterium cf. prausnitzii KLE1255 |  | Veillonella parvula |
| Paraprevotella clara YIT 11840 |  | Bifidobacterium breve |
| Coprococcus comes ATCC 27758 |  | Bifidobacterium pseudocatenulatum |
| Dorea longicatena AGR2136 |  | Collinsella aerofaciens |
| Ruminococcus bromii L2-63 |  | Bacteroides dorei |
| Alistipes shahii WAL 8301 |  | Bacteroides xylanisolvens |
| Prevotella copri DSM 18205 |  | Ruminococcus gnavus |
| Dialister invisus DSM 15470 |  | Rothia mucilaginosa |
| Ruminococcus gnavus ATCC 29149 |  | Erysipelatoclostridium ramosum |
| Barnesiella intestinihominis YIT 11860 |  | Flavonifractor plautii |
| Adlercreutzia equolifaciens DSM 19450 |  | Clostridium innocuum |
| Bilophila wadsworthia ATCC 49260 |  | Bacteroides thetaiotaomicron |
| Alistipes ihumii AP11 |  | Anaerostipes hadrus |
|  |  | Blautia wexlerae |
|  |  | Acidaminococcus intestini |
|  |  | Bifidobacterium bifidum |
|  |  | Bacteroides cellulosilyticus |
|  |  | Parabacteroides distasonis |
|  |  | Lachnospira pectinoschiza |
|  |  | Enterococcus faecium |
|  |  | Alistipes putredinis |
|  |  | Veillonella seminalis |
|  |  | Eggerthella lenta |
|  |  | Haemophilus parainfluenzae |
|  |  | Sellimonas intestinalis |
|  |  | Escherichia marmotae |
|  |  | Roseburia intestinalis |
|  |  | Odoribacter splanchnicus |
|  |  | Prevotella copri |
|  |  | Parabacteroides merdae |
|  |  | Bacteroides nordii |
|  |  | Bacteroides caccae |
|  |  | Streptococcus mitis |
|  |  | Bifidobacterium dentium |
|  |  | Gordonibacter pamelaeae |
|  |  | Bacteroides stercoris |
|  |  | Clostridium bolteae |
|  |  | Akkermansia muciniphila |
|  |  | Prevotella buccae |
|  |  | Streptococcus vestibularis |
|  |  | Streptococcus infantis |
|  |  | Veillonella dispar |
|  |  | Bacteroides finegoldii |
|  |  | Bacteroides sp. CAG 443 |
|  |  | Sutterella parvirubra |
|  |  | Collinsella intestinalis |
|  |  | Collinsella stercoris |
|  |  | Bacteroides faecis |
|  |  | Bilophila wadsworthia |
|  |  | [Collinsella] massiliensis |
|  |  | Bacteroides salyersiae |
|  |  | Phascolarctobacterium faecium |
|  |  | Clostridium clostridioforme |
|  |  | Bacteroides faecis CAG 32 |
|  |  | Enorma massiliensis |
|  |  | Parabacteroides goldsteinii |
|  |  | Parabacteroides johnsonii |
|  |  | Phascolarctobacterium succinatutens |
|  |  | Klebsiella michiganensis |
|  |  | Veillonella infantium |
|  |  | Bifidobacterium catenulatum |
|  |  | Eubacterium eligens |
|  |  | Roseburia inulinivorans |
|  |  | Ruminococcus torques |
|  |  | Paraprevotella xylaniphila |
|  |  | Staphylococcus epidermidis |
|  |  | Bacteroides eggerthii |
|  |  | Slackia isoflavoniconvertens |
|  |  | Aeriscardovia aeriphila |
|  |  | Coprobacter fastidiosus |
|  |  | Ruminococcus bromii |
|  |  | Megamonas funiformis |
|  |  | Megamonas funiformis CAG 377 |
|  |  | Megamonas hypermegale |

**Supplementary Table 3. Maternal niche-associated bacteria contributing to infant gut microbiome based on structural variations analysis in the multiple ecological niche cohort (n = 25 pairs).**

| **Fecal** | **Skin** | **Vaginal** |
| --- | --- | --- |
| Bacteroides uniformis ATCC 8492 | Bacteroides uniformis ATCC 8492 | Bacteroides uniformis ATCC 8492 |
| Alistipes putredinis DSM 17216 | Alistipes putredinis DSM 17216 | Alistipes putredinis DSM 17216 |
| Parabacteroides distasonis ATCC 8503 | Barnesiella intestinihominis YIT 11860 | Parabacteroides distasonis ATCC 8503 |
| Prevotella copri DSM 18205 | Blautia wexlerae DSM 19850 | Prevotella copri DSM 18205 |
| Ruminococcus torques L2-14 | Bacteroides coprocola DSM 17136 |  |
| Blautia wexlerae DSM 19850 | Ruminococcus bromii L2-63 |  |
| Barnesiella intestinihominis YIT 11860 | Bacteroides massiliensis B84634 = Timone 84634 = DSM 17679 = JCM 13223 |  |
| Bifidobacterium bifidum PRL2010 | Bifidobacterium bifidum PRL2010 |  |
| Roseburia hominis A2-183 | Blautia obeum ATCC 29174 |  |
| Bacteroides coprocola DSM 17136 | Akkermansia muciniphila ATCC BAA-835 |  |
| Parabacteroides merdae ATCC 43184 | Alistipes shahii WAL 8301 |  |
| Blautia obeum ATCC 29174 | Bacteroides xylanisolvens XB1A |  |
| Dorea longicatena AGR2136 | Ruminococcus torques L2-14 |  |
| Bacteroides xylanisolvens XB1A | Dorea longicatena AGR2136 |  |
| Coprococcus comes ATCC 27758 | Coprococcus comes ATCC 27758 |  |
| Akkermansia muciniphila ATCC BAA-835 | Dorea formicigenerans 4_6_53AFAA |  |
| Ruminococcus bromii L2-63 | Roseburia hominis A2-183 |  |
| Bacteroides massiliensis B84634 = Timone 84634 = DSM 17679 = JCM 13223 | Faecalibacterium cf. prausnitzii KLE1255 |  |
| Dorea formicigenerans 4_6_53AFAA |  |  |
| Alistipes shahii WAL 8301 |  |  |
| Faecalibacterium cf. prausnitzii KLE1255 |  |  |
| Bacteroides caccae ATCC 43185 |  |  |
| Bifidobacterium pseudocatenulatum DSM 20438 = JCM 1200 = LMG 10505 |  |  |
| Paraprevotella clara YIT 11840 |  |  |
| Sutterella wadsworthensis HGA0223 |  |  |
| Holdemanella biformis DSM 3989 |  |  |
| Ruminococcus lactaris ATCC 29176 |  |  |
| Methanobrevibacter smithii ATCC 35061 |  |  |
| Eubacterium ventriosum ATCC 27560 |  |  |
| Roseburia intestinalis XB6B4 |  |  |
| Coprococcus catus GD/7 |  |  |
| Phascolarctobacterium succinatutens YIT 12067 |  |  |

**Supplementary Table 4.** **Maternal niche-associated bacteria contributing to infant oral microbiome based on structural variations analysis in the multiple ecological niche cohort (n = 25 pairs).**

| **Fecal** | **Skin** | **Vaginal** |
| --- | --- | --- |
| Bacteroides uniformis ATCC 8492 | Bacteroides uniformis ATCC 8492 | Prevotella copri DSM 18205 |
| Faecalibacterium cf. prausnitzii KLE1255 | Ruminococcus bromii L2-63 |  |
| Ruminococcus bromii L2-63 | Faecalibacterium cf. prausnitzii KLE1255 |  |
| Prevotella copri DSM 18205 | Blautia wexlerae DSM 19850 |  |
| Parabacteroides distasonis ATCC 8503 | Alistipes putredinis DSM 17216 |  |
